# A rare case report of triple primary malignancies: synchronous breast ductal invasive carcinoma and lung neuroendocrine tumor, followed by chronic myeloid leukemia

**DOI:** 10.1016/j.ijscr.2025.111305

**Published:** 2025-04-14

**Authors:** Iraj Feizi, Atabak Sedigh-Namin, AmirAbbas Kani, Sonia Sharifi Namin, Alireza Bagheri Toularoud

**Affiliations:** a Department of Surgery, School of Medicine, Ardabil University of Medical Sciences, Ardabil, Iran; b Students Research Committee, School of Medicine, Ardabil University of Medical Sciences, Ardabil, Iran; c SHAFA PARTOO ARDEBIL Medical Center, Radiational Oncology Department, Ardabil, Iran; d Department of Anatomical Sciences and Pathology, School of Medicine, Imam Reza Hospital, Ardabil University of Medical Sciences, Ardabil, Iran

**Keywords:** Invasive ductal carcinoma, Colon, Neuroendocrine tumor, Chronic granulocytic leukemia, Case report

## Abstract

**Introduction and importance:**

Multiple primary malignancies (MPMTs), the occurrence of two or more different primary cancers in a single person, are rare. These tumors can be synchronous or metachronous, with an incidence ranging from 0.73 % to 11.70 % in cancer patients. While invasive ductal carcinoma (IDC) is the most common form of breast cancer and lung neuroendocrine tumors (NETs) are rare, their co-occurrence as MPMT is extremely rare. In addition, chronic myeloid leukemia (CML) makes such cases even more complex.

**Case presentation:**

We report the case of a 59-year-old woman who presented with a lump in the left breast. Diagnostic examinations confirmed breast IDC. Metastatic examination identified a separate primary, well-differentiated NET of the left lung. Both malignancies were treated surgically followed by appropriate adjuvant therapy. A year later, routine follow-up revealed an elevated white blood cell count and a subsequent bone marrow biopsy confirmed the diagnosis of CML.

**Clinical discussion:**

The diagnosis of IDC and pulmonary NET as primary tumors is rare and the subsequent development of CML in this setting has not been reported previously. This case highlights the critical importance of thorough diagnostic evaluations to accurately differentiate between metastatic disease and MPMTs. The occurrence of three different malignancies in a single patient presents significant treatment challenges and highlights the need for personalized treatment approaches.

**Conclusion:**

This report emphasizes the need for comprehensive diagnostic protocols in the evaluation of multiple tumors and contributes to the growing body of knowledge about MPMT. The coexistence of IDC, pulmonary NET and subsequent CML represents a rare and complex clinical scenario that requires continuous research into optimal management strategies for such cases.

## Highlights

• Multiple Primary Malignant Tumors (MPMTs) are rare, with synchronous or metachronous presentations occurring in 0.73%-11.70% of cancer patients.
• This case describes an unusual combination of invasive ductal carcinoma (IDC) of the breast, a lung neuroendocrine tumor (NET), and subsequent chronic myeloid leukemia (CML).
• Diagnostic evaluations distinguished IDC and NET as independent primary malignancies, confirmed by histopathology and immunohistochemical analysis.
• The development of CML during follow-up highlights the complexity of managing triple malignancies and the importance of personalized treatment plans.
• This report underscores the necessity of thorough diagnostic workups to differentiate between metastatic disease and MPMTs, contributing to the understanding of these rare clinical scenario

## 1 Introduction

This case report follows the SCARE criteria exclusively to ensure a structured and comprehensive presentation of the clinical scenario [1]. Multiple Primary Malignant Tumors (MPMTs) defined as two or more primary cancers in one patient. These tumors can be synchronous (discovered within six months) or metachronous (discovered after six months). MPMT are uncommon, affecting between 0.73 % and 11.70 % of cancer patients [2]. The main objective of this case report is to explore the rare co-occurrence of multiple primary malignancies, with a focus on the prevalence and pathophysiology of MPMTs**.** A rare type of lung cancer is called a neuroendocrine tumor (NET). Though they can develop in many other regions of the body, lung cancer causes 1–2 % of all lung cancers and around 25 % of all NETs, making the lung the second most common site for these tumors [3]. Invasive ductal carcinoma (IDC) is the most prevalent type of breast cancer, comprising roughly 75 % of all breast cancer diagnoses [4]. CML (Chronic granulocytic leukemia) is a slow-progressing blood and bone marrow disease that typically develops in middle age or later, and rarely affects children. In Western countries, CML accounts for 15–25 % of all adult leukemias and 14 % of all leukemias [6].

While the co-occurrence of IDC and NET often indicates a metastatic relationship, their coexistence as MPMTs is rare. Case reports on MPMTs are increasingly valuable due to their rising prevalence. Advancements in screening and diagnostic technologies have made early detection of these malignancies more feasible, making case reports essential in understanding the complexities of MPMTs. However, our case report presents an unusual clinical scenario: a patient diagnosed with both primary breast cancer and primary lung cancer following a CT scan prompted by respiratory symptoms. The breast cancer was confirmed as a separate primary tumor. After further follow-up, a diagnosis of CML was made, and it was added to the patient's two initial cancers. This case challenges the conventional understanding of MPMTs, highlighting their complexity and the difficulty in treatment planning. The aim of this report is to document this rare occurrence and contribute to the growing body of knowledge on MPMTs.

## 2 Case report

A 59-year-old female patient, a non-smoker, with a medical history of hypothyroidism treated with daily levothyroxine, presented with a lump in her left breast that she noticed approximately a month ago. The mass had been palpable for about a month, and its size had not increased rapidly. The patient reported no breast pain, nipple discharge, or weight loss in recent months. She also had no family history of similar diseases.

**Fig. 1:**
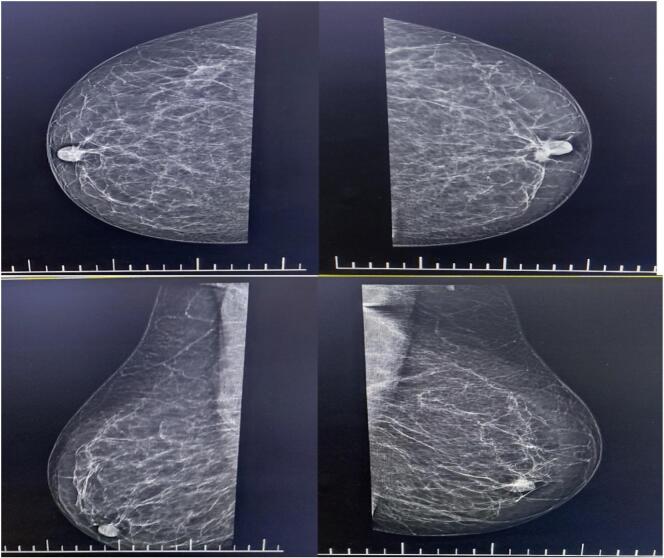
A mammogram shows bilateral breast composition type A with a BI-RADS 4 assessment for the left breast and BI-RADS 1 for the right; axillary lymph nodes are unremarkable.

Physical examination revealed no asymmetry, nipple inversion, nipple discharge, or skin abnormalities in the left breast. No signs of congestion or erythema were noted. However, a firm, fixed mass was palpated at the 11 o'clock position near the nipple. Based on these findings, a mammogram was recommended. The mammogram revealed bilateral breast composition A (Fig. 1). An isoechoic mass with irregular borders, measuring 12 × 8 mm, was observed near the left nipple. Following this, a targeted ultrasound revealed a hypoechoic mass measuring 7 × 4.5 mm, non-parallel in orientation, with irregular margins, located at the 10 o'clock position approximately 7 mm from the skin surface. The BI-RADS assessment for the left breast was 4, while the right breast had a BI-RADS score of 1. Both axillary lymph nodes were unremarkable.

**Fig. 2:**
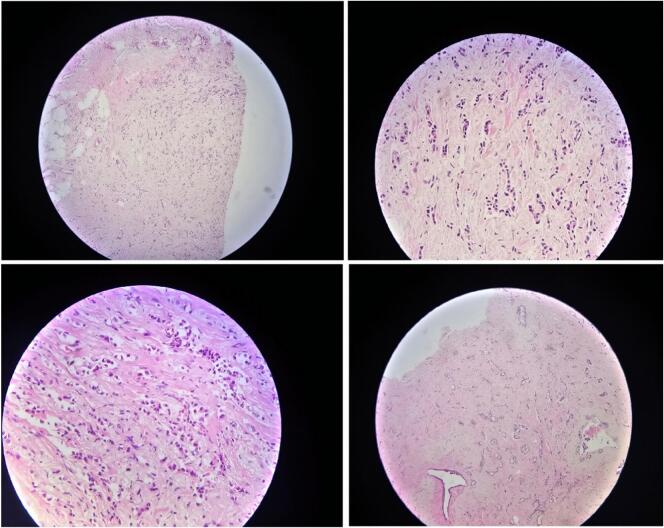
Invasive ductal carcinoma, NOS type, infiltrative small nests and occasional tubules about 10 % (score3) with moderate enlarged nuclei(score2), scattered mitoses about 2 points in 10hpf (score 1)within desmoplastic stroma.

A core needle biopsy revealed invasive ductal carcinoma (NST), moderately differentiated (grade 7/9) based on the Nottingham scoring system. The tumor exhibited poorly formed tubules, intermediate nuclear features, and moderate mitotic activity. No pre-cancerous lesions, local spread, or tumor necrosis were identified (Fig. 2). Immunohistochemical staining was positive for CK and showed patchy positivity for TTF-1, while GATA-3, HER-2, ER, and PR were negative. Ki-67 expression was low at 1 %. Metastatic breast carcinoma was excluded based on these findings. Following breast-conserving surgery (BCS) and axillary lymph node dissection (ALND), the patient was discharged in good condition. Pathology of the resected breast tissue and lymph nodes confirmed ductal carcinoma, with margins free of malignant cells, and all 9 lymph nodes were negative for malignancy. Thirty sessions of hormone therapy were initiated as adjuvant treatment.

**Fig. 3:**
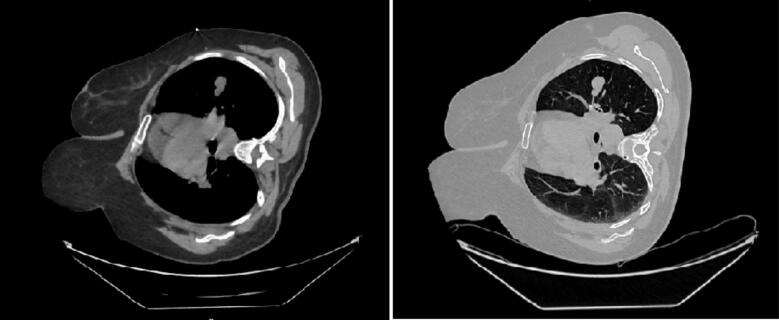
Chest spiral CT reveals a 17 × 25 mm lobulated mass in the left lung apex with no invasion into surrounding tissues.

**Fig. 4:**
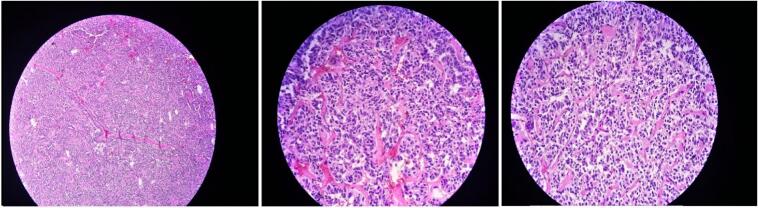
Pulmonary typical carcinoid tumor, showing solid nests of cells with a zellballen pattern, trabecullar and pseudoglandular pattern.

A chest spiral CT scan was performed to evaluate for metastasis, which revealed a mass in the left lung apex measuring 17 × 25 mm with a well-defined, lobulated border and no invasion into surrounding tissues (Fig. 3). A core needle biopsy of the left lung mass confirmed a well-differentiated neuroendocrine tumor (carcinoid) of grade I (Fig. 4), composed of small cells within a loose fibrovascular stroma. A bone scan did not reveal any evidence of metastasis.

The patient has been undergoing regular follow-up assessments following her breast cancer treatment. Two CT scans have been performed, neither of which showed any signs of lymph node involvement or metastatic spread. Furthermore, the left lung nodule has remained stable in size. A bilateral mammogram showed normal findings, with a BI-RADS score of 2 for the left breast and 1 for the right breast, indicating a predominantly fatty composition (BI-RADS density A) without palpable masses or nodal involvement. Serial follow-up ultrasound examinations showed no evidence of breast cancer recurrence in either the breast tissue or axillary lymph nodes.

One year later, the patient underwent thoracotomy with wedge resection of the left lung mass. Following the surgery, the patient was discharged after completing her postoperative care. She received adjuvant therapy, including a daily dose of 2.5 mg Letrozole, calcium supplements daily, and one Alendronate tablet weekly to prevent osteoporosis.

**Fig. 5:**
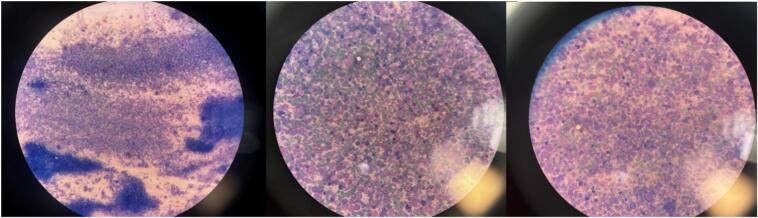
Bone marrow biopsy, bone particles and hypercellular marrow with absolute myeloid hyperplasia, left shifted with 2 peaks: myelocytes and segmented neutrophils, increased eosinophils and basophils. No significant granulocytic dysplasia.

During a routine follow-up one year later, the patient's white blood cell (WBC) count was found to be elevated at 143,000. A bone marrow biopsy (Fig. 5) revealed hypercellular marrow with absolute myeloid hyperplasia, showing a left shift with two peaks: myelocytes and segmented neutrophils, along with increased eosinophils and basophils. No significant granulocytic dysplasia was present. Based on these findings, the patient was diagnosed with Chronic Myelogenous Leukemia (CML) and began treatment with Imatinib.

## 3 Discussion

Breast cancer and lung cancer are among the most prevalent malignancies worldwide, frequently ranking among the top causes of cancer incidence. Invasive ductal carcinoma (IDC) is the most common type of breast cancer, accounting for approximately 75 % of all cases. Lung neuroendocrine tumors (LNETs) are relatively rare, with limited data available regarding their incidence and prevalence. Pulmonary carcinoids, a subtype of LNET, occur at a rate of approximately 0.2 to 2 cases per 100,000 population in the United States and the European Union. In this report, we present a unique case of a patient diagnosed with both lung NET and IDC of the breast, a combination not previously documented in the literature. The patient's case is further distinguished by the development of chronic myeloid leukemia (CML) during follow-up, making this case a rare occurrence of triple primary cancers. This unusual combination of malignancies highlights the complexity of diagnosis and treatment, especially in the absence of advanced imaging techniques like PET-CT, which are often used to detect metastasis or aid in distinguishing between primary tumors.

When diagnosing double primary carcinoma, it is crucial to rule out the possibility of metastatic disease. The lungs are a common site for metastasis in patients with breast cancer [9]. Previous reports have typically involved metastatic spread from the lung to the breast or vice versa, or different types of cancers developing. Distinguishing between two primary tumors and metastatic disease can often be challenging. In our case, both the breast and lung tumors were confirmed as primary malignancies through pathological and immunohistochemical examinations. Furthermore, the third malignancy, CML, was diagnosed during the patient's follow-up while receiving hormone therapy. The lack of advanced imaging like FDG PET or DOTATATE PET CT made it difficult to assess the full scope of metastatic spread, which could have provided more clarity in the treatment planning.

A retrospective cohort study examining metachronous second primary cancers found that the uterus, ovary, and thyroid were the most common sites for second primary cancers after an initial breast cancer. Additionally, the thyroid, larynx, and oral/pharyngeal regions were common sites following an initial lung cancer [10]. A 20-year study in Queensland confirmed that lung cancer patients had a higher risk of developing esophageal and head and neck cancers compared to the general population [11]. However, in our patient, the second primary cancer did not originate from the commonly reported sites. This highlights that there is no definitive correlation for site predilection in double primary malignant tumors. Therefore, the possibility of multiple primary cancers should be considered in patients with multiple tumors. In this case, the rarity of the lung NET and IDC co-occurring highlights the need for more research into the underlying mechanisms of multiple primary malignancies and the specific risk factors that may contribute to their development.

Bone metastases (BM) are common complications of lung and breast cancers, associated with poor prognosis and high mortality rates. Current treatment options remain limited in their effectiveness [12]. Approximately 40 % of patients with advanced adenocarcinoma and 50 % of patients with small cell lung cancer (SCLC) develop BM [13]. At diagnosis, BM are present in 18 % of SCLC cases, rising to 50–65 % within two years [14]. Median survival for patients with SCLC and BM is only 4.9 months [15]. Breast cancer ranks second in terms of BM incidence, with rates ranging from 10 to 30 % [16]. BM typically develop 2–3 years after the initial breast cancer diagnosis [17]. The small size, limited adhesion, and early metastasis of SCLC contribute to BM formation [18,19]. In our case, two years later, the patient presented with an elevated WBC count, and bone marrow examination confirmed the diagnosis of CML. Although bone metastasis was not present in this case, the progression to CML highlighted the importance of monitoring for additional malignancies in patients with multiple cancers.

There is currently no established, consistent clinical treatment guideline for synchronous primary cancers [20]. Surgery remains the primary therapeutic approach for managing such cases. In the absence of standardized treatment protocols for this complex scenario, the management plan had to be adapted to the individual patient's needs, with regular follow-up assessments and careful monitoring for additional malignancies.

In conclusion, this manuscript presents a rare case of a patient diagnosed with both a lung NET and IDC of the breast, with a subsequent diagnosis of CML during adjuvant therapy. While such cases are uncommon, when imaging studies reveal a mass in another organ, the possibility of a new primary tumor should be carefully considered rather than assuming it is metastasis from the original cancer. A combination of tumor marker analysis, imaging findings, and clinical characteristics can assist in making a preoperative diagnosis, but the final diagnosis must be confirmed through pathological and immunohistochemical examination. Given the absence of advanced imaging tools in this case, further investigation is needed to establish effective treatment protocols and to refine diagnostic strategies for identifying multiple primary malignancies.

## 4 Conclusion

In our case, we initially considered the breast malignancy to be a primary cancer with lung metastasis. However, after a thorough diagnostic workup, including histopathological and immunohistochemical analysis, we confirmed the presence of two distinct primary malignancies: invasive ductal carcinoma of the breast and a well-differentiated neuroendocrine tumor of the lung. Subsequently, during follow-up, the patient was diagnosed with chronic myeloid leukemia, adding complexity to the clinical scenario. This rare combination of triple primary cancers highlights the critical importance of comprehensive evaluation in patients with multiple tumors to accurately distinguish between metastatic disease and multiple primary malignancies. Moreover, it emphasizes the need for further research into the underlying mechanisms and risk factors associated with such rare occurrences.

## CRediT authorship contribution statement

Atabak Sedigh-namin: Data collection, data analysis, and manuscript writing, and first author.

Alireza Bagheri toularoud: Study design, data interpretation, and manuscript review, and first author.

Iraj feizi: Study concept and design, data interpretation, and manuscript review, and corresponding author.

AmirAbbas Kani: Data interpretation.

Sonia Sharifi Namin: Data interpretation.

## Consent

Written informed consent was obtained from the patient for publication of this case report and accompanying images. A copy of the written consent is available for review by the Editor-in-Chief of this journal on request.

## Ethical approval

This study was conducted in accordance with the ethical principles outlined in the Research Ethics Committees of Ardabil University of Medical Sciences. As this research, it was determined to be exempt from formal ethical review by the Research Ethics Committees of Ardabil University of Medical Sciences.

The study was reviewed and approved by the relevant Research Ethics Committee in accordance with ethical guidelines.

## Guarantor

Alireza Bagheri toularoud accepts full responsibility for the work and the conduct of the study, had access to the data, and controlled the decision to publish.

## Sources of funding

None.

## Registration of research studies

None.

## Declaration of competing interest

N/A.
